# P-Type Lithium Niobate Thin Films Fabricated by Nitrogen-Doping

**DOI:** 10.3390/ma12050819

**Published:** 2019-03-11

**Authors:** Wencan Li, Jiao Cui, Weiwei Wang, Dahuai Zheng, Longfei Jia, Shahzad Saeed, Hongde Liu, Romano Rupp, Yongfa Kong, Jingjun Xu

**Affiliations:** 1 The MOE Key Laboratory of Weak-Light Nonlinear Photonics and TEDA Institute of Applied Physics, Nankai University, Tianjin 300457, China; 1120160065@mail.nankai.edu.cn (W.L.); cj19890823@126.com (J.C.); jialf666@163.com (L.J.); shehzadsaeed2003@yahoo.com (S.S.); romano.rupp@univie.ac.at (R.R.); 2 School of Physics, Nankai University, Tianjin 300071, China; weiweiwang@mail.nankai.edu.cn (W.W.); liuhd97@nankai.edu.cn (H.L.); 3 Faculty of Physics, University of Vienna, 1090 Vienna, Austria

**Keywords:** lithium niobate film, pulsed laser deposition, nitrogen-doped, p-type conductivity

## Abstract

Nitrogen-doped lithium niobate (LiNbO_3_:N) thin films were successfully fabricated on a Si-substrate using a nitrogen plasma beam supplied through a radio-frequency plasma apparatus as a dopant source via a pulsed laser deposition (PLD). The films were then characterized using X-Ray Diffraction (XRD) as polycrystalline with the predominant orientations of (012) and (104). The perfect surface appearance of the film was investigated by atomic force microscopy and Hall-effect measurements revealed a rare p-type conductivity in the LiNbO_3_:N thin film. The hole concentration was 7.31 × 10^15^ cm^−3^ with a field-effect mobility of 266 cm^2^V^−1^s^−1^. X-ray Photoelectron Spectroscopy (XPS) indicated that the atom content of nitrogen was 0.87%; N atoms were probably substituted for O sites, which contributed to the p-type conductivity. The realization of p-type LiNbO_3_:N thin films grown on the Si substrate lead to improvements in the manufacturing of novel optoelectronic devices.

## 1. Introduction

Lithium niobate (LiNbO_3_, LN), as an important and versatile crystal, is famous for its excellent piezoelectric, thermoelectric, nonlinear optical, and electro-optical properties [1,2,3,4,5,6,7,8,9,10]. LN doped with different elements presents special properties and it is the best crystal of the composite index with a wide market application, as exemplified by ferroelectrics [1], nonlinear photonic crystal [3,4,5], photodetector [7], electro-optical modulator [9,10,11], optical waveguide [8,12], and so on. However, these applications act as passive components. LN is rarely reported as an active component. If passive and active components were integrated on one LN substrate, a real all-light photonic chip would be accomplished. To fabricate an active component, the basic unit is the p-n junction. The carrier type of LN is greatly affected by the dopant and oxidation-reduction states of the crystal. Unfortunately, LN grown with conventionally-doped elements usually exhibits n-type conductivity [13,14], which has made it very difficult to manufacture stable and strong p-type LN materials.

In fact, tremendous efforts have been focused on the fabrication of a p-type LN. It was reported that the majority of carriers are holes for undoped LN when the crystal was irradiated by ultraviolet light [15]. Recent results show that the majority of carriers of LN:Zr [16] and LN:Hf [17] are still electrons, even in the ultraviolet region. Although the photoinduced hole carrier concentration of heavily doping-Mg LiNbO_3_ (up to 4.6%) is nearly equal to the electron concentration [18], the majority of carriers are still not holes. It was reported that the majority carriers change to holes by increasing Zn content up to 8%, which is above its concentration threshold [19]. The tendency is just the same with LiNbO_3_:Mg [18] and the concentration of holes as majority carriers is still so low that it cannot be detected by Hall-effect measurements. Recently, Pei et al. investigated various doped and oxidized LN crystals and found that it is difficult to obtain p-type LN by highly doping optical damage resistant ions into the crystal and the only possible way may be the thermo-electric oxidization method [20]. However, after strong thermo-electric oxidization, the crystal quality is bad because of the heavy out-diffusion of lithium, which limits the practical application of this technique. 

For another famous oxide crystal, ZnO, it is also difficult to make p-type samples because of the zinc or hydrogen interstitials and potential oxygen vacancies, causing the material to be naturally n-type [21,22]. It was reported that ZnO doped by nitrogen (ZnO:N) can achieve p-type conductivity [23,24,25,26]. Therefore, an interesting question arises: What role will N will play if it is doped into LN? However, when attempting to grow LN crystals doped with nitride, such as Si_3_N_4_ or BN, we found that N element could not be detected in the as-grown LN crystals using X-ray Photoelectron Spectroscopy (XPS), which means that these nitrides are not stable in the LN melt and N^3−^ ions were oxidized. We also attempted to grow LN thin films under the nitrogen atmosphere, but no N ion was found in the films.

In this work, we introduce a novel method that provides a one-step fabricating p-type LiNbO_3_:N films on a Si (111) substrate by combining pulsed laser deposition and radio frequency (PLD-RF) technique. Hole mobility (266 cm^2^V^−1^s^−1^) and concentration (7.31 × 10^15^ cm^−3^) of the film were measured via the Hall-effect. The atomic content of nitrogen is shown to be 0.87% via XPS. The results indicate that a film with p-type conduction was successfully fabricated and it can open a new tool box for the fabrication of integrated devices based on LN thin films.

## 2. Experimental Details

### 2.1. Preparation of LN:N thin films

Nitrogen-doped LN (LN:N) thin films were deposited on Si (111) substrates using the pulsed laser deposition (PLD) technique with a 248 nm KrF excimer from Coherent assisted using RF nitrogen plasma. The SiO_2_ layer on the surface of Si substrate was removed by placing it in HF solution (about 3%) for 1 min. Then the substrate was cleaned for 15 min in the alcohol ultrasonically and rinsed with deionized water for further cleaning. Subsequently, it was immediately put into a vacuum chamber. We choose a c-oriented congruent LN (CLN) crystal as the target and set the laser pulses conditions that the fluence is 1.5 J/cm^2^ and the repetition rate is 3 Hz. The target-substrate distance was 40 mm. During the deposition processing, the substrate temperature was kept at 750 °C. 

After deposition for one hour, the films were annealed under the oxygen atmosphere for 1 h and then naturally cooled to room temperature. We selected three related parameters (Pressure, Gas flow, and RF current) to adjust the optimal conditions for growing the films. Table 1 shows the fabrication details of different films. 

### 2.2. Instrumentation details

X-ray diffraction (XRD) patterns were measured using a Philips X’ Pert PRO X-ray Diffractometer (Philips Japan, Ltd. Tokyo, Japan) (Cu Kα, λ = 1.54056 Å). The surface morphology of the film was examined using an MFP-3D Infinity atomic force microscope (Oxford Instruments plc, Oxon, UK). Hall-effect experiments were performed at 300 K using a HL5550PC Hall effect measurement system (Accent optical technologies company, Bend, USA). XPS measurements were carried out with an ESCA Lab 220i-XL spectrometer using a non-monochromatic Al Kα (1486.6 eV) X-ray source (Kratos Analytical Ltd., Manchester, UK). All of the spectra were calibrated to the binding energy of the adventitious C1s peak at 284.6 eV.

## 3. Results and Discussion

### 3.1. Structure of LN:N Films

**Figure 1:**
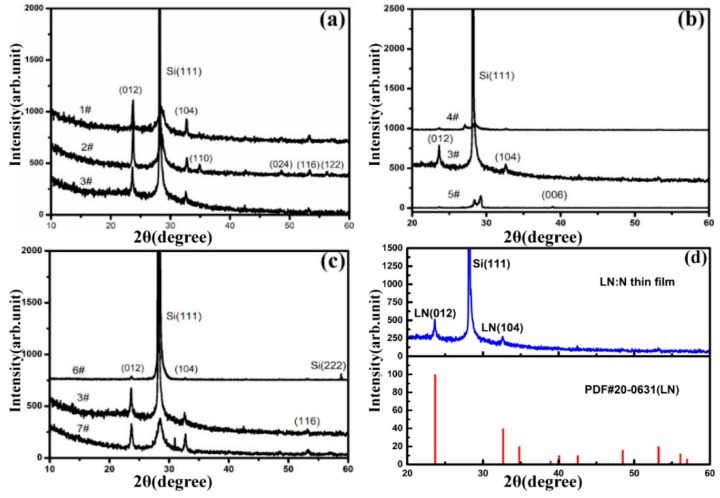
X-ray diffraction (XRD) patterns and phase analyses of LN:N thin films. Fabricated (**a**) with different N_2_ pressures: (1#) 43 Pa, (2#) 53 Pa, (3#) 70 Pa; (**b**) with different gas (N_2_) flows: (4#) 45 sccm, (3#) 87 sccm, (5#) 100 sccm; (**c**) with different RF currents: (6#) 12 mA, (3#) 18 mA, (7#) 20 mA; (**d**) XRD pattern of LN:N film fabricated with ideal conditions (70 Pa, 87 sccm, 18 mA) and PDF card of LiNbO_3_.

We successfully fabricated the LN films using the PLD method with different experimental conditions, i.e. applying different the pressure of N_2_, gas flows (N_2_) and RF currents. The XRD patterns of the films are shown in Figure 1. As shown in Figure 1a, the samples 1#, 2#, and 3# were fabricated with different N_2_ pressure (Table 1). We can see that thin films 1# and 2# show diffraction peaks at (116), (110), (024), and (112), while only two diffraction peaks of 3# thin film were observed at 2θ = 23.7° and 32.7°, corresponding to the orientations (012) and (104). The result indicates a better structural quality of sample 3# than that of 1# and 2#. Compared to sample 3#, we deposited 4# and 5# thin films with various gas flow at 45 sccm and 100 sccm, respectively (Table 1). As shown in Figure 1b, there are no obvious peaks of LiNbO_3_ for samples 4# and 5#. Figure 1c shows the diffraction patterns of LN:N thin films fabricated by different RF current of 12 mA (6#), 18 mA (3#), and 20 mA(7#) (Table 1), respectively. We can find that sample 6# shows very weak peaks of LN and there are obvious hybrid peaks for sample 7# caused by a high RF current. All of these experiments indicate that 70 Pa N_2_ pressure, 87 sccm gas flow, and 18 mA RF current are an ideal set of experimental fabrication conditions for deposition of LN:N thin films.

Based on the results, the collision between the particles sputtering out of the target material and the gas molecules is more appropriate in case of suitable pressure, RF current, and gas flow, which results in better crystalline of particles when they reach the substrate surface. Under the conditions of sample 3# (70 Pa, 87 sccm, 18 mA), another superior LN:N thin film was successfully fabricated, Figure 1d shows XRD of LN:N film. There are only two reflection peaks at 2θ = 23.7° and 32.6°, which are assigned to the (012) and (104) peaks of LN compared with the standard card (PDF#20-0631), whereas the other diffraction peak at 2θ = 28.2° corresponds to the Si (111) substrate. The result proves that the film is well polycrystalline with the predominant orientations of (012) and (104).

**Figure 2:**
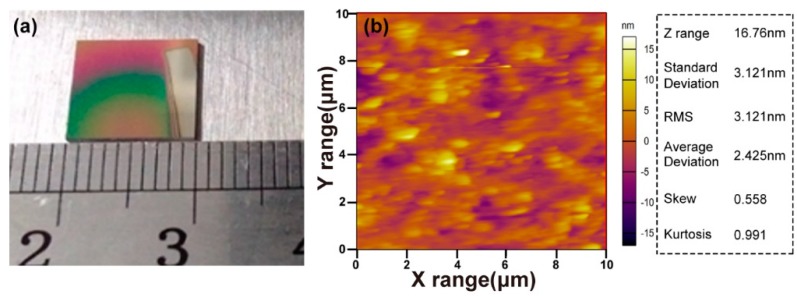
(**a**) The LN:N film fabricated with ideal conditions (70 Pa, 87 sccm, 18 mA) and (**b**) the 2D topographic AFM image taken from a (10 × 10) μm^2^ scan area of the LN:N film. The root mean square roughness (RMS) is 3.12 nm.

Figure 2a shows a thin film sample fabricated under ideal conditions on the Si-substrate (Figure 1d XRD), of which the square is 10.0 mm × 10.0 mm. The sparsely color difference, due to the diffraction, suggests that the thickness of the film is topo-homogeneous. The surface morphology and smoothness of the films were investigated by atomic Force Microscope (AFM). The 2D topographic AFM image is presented in Figure 2b. The root-mean-square roughness (RMS) is 3.12 nm and the Z range shows us that the height is 16.76 nm. Consequently, these results indicate that LN thin film doped N-element were deposited successfully and it was well polycrystalline.

### 3.2. Electrical Characterization of LN:N film

#### 3.2.1. Hall-Effect Measurement

Several methods such as the Hall Effect, Fanning Scattering, and Holography [27] can be used to judge the carrier type (n or p) and to study the electrical conduction performance of LN:N thin films. Carrier concentration, resistivity, and mobility were measured by Hall-effect experiments, which were carried out at 300 K. 

**Figure 3:**
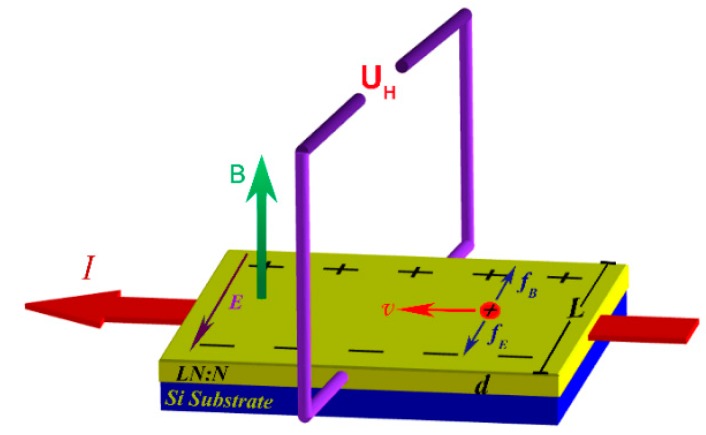
Schematic diagram of the Hall-effect measurement. The current **I** passes through the thin film, **B** is the magnetic field, **d** is the thickness of the film, **L** is the width of the thin film, and **U_H_** is the hall voltage.

Figure 3 shows the schematic of the Hall-effect measurement. The magnetic field B passes vertically through the sample, while the current I flows past the film from the one side to the other. The properties can be obtained by testing the Hall voltage U_H_. Electrons drifting under the Hall-effect in equilibrium is described by the following equations:

(1) q∗v∗B=q∗E

(2) $$U_{H}=E*L=v*B*L$$

(3) I=n∗q∗v∗s

where *q* is the charge of the charge carrier, *v* is the drift velocity of a carrier, *n* is a carrier concentration, and s=d∗L. From the Equations (1)–(3), we can get:

(4) $$U_{H}=(I*B)/(n*q*d)=K*I*B/d$$

The Hall coefficient is:

(5) K=1/(n∗q)

And the carrier concentration:

(6) n=1/(K∗q)

In the follow equation, here *σ* is the conductivity and *μ* is the mobility:

(7) σ=nqμ

Then we can get:

(8) μ=kσ

From the Equations (1)–(8), the positive Hall coefficient of the LN:N film is +853 cm^3^/C, which proves that the carrier charge *q* is positive and the majority carriers are holes. The conductivity at room temperature is 0.311 Ω^−1^cm^−1^, which increased by ten orders of magnitudes compared with previous reports [28,29]. The carrier concentration is 7.31 × 10^15^ m^−3^ and the mobility is up to 266 cm^2^V^−1^s^−1^. All of the results demonstrate an excellent p-type conductivity characteristic of LN:N thin films. The realization of p-type nitrogen-doped LN films grown on the Si substrate will lead to improvement in the fabrication of novel optical-electronic devices.

#### 3.2.2. XPS measurement

The high-resolution XPS spectra of the film surface was measured to determine the nitrogen chemical composition in the LN:N films. The spectrum of the N1s region, which covers the binding energy of N1s electrons, was analyzed using the Gaussian fitting function.

**Figure 4:**
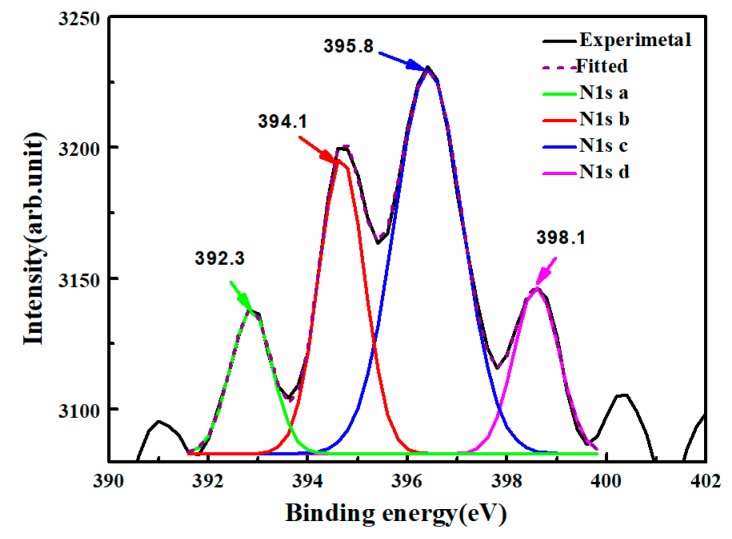
XPS spectra recorded from a LN:N film grown by PLD-RF. The spectrum was analyzed using the Gaussian fitting function. There are four peaks at 392.3, 394.1, 395.9, and 398.1 eV.

As shown in Figure 4, there are four peaks at 392.3, 394.1, 395.9, and 398.1 eV. According to the previous report, the NO^−^ or NO_2_^−^ type species should appear above 400 eV [30], so the peaks appearing at 392.3, 394.1, 395.9, and 398.1 eV can be attributed to the anionic N^−^. As we know, there are Li-vacancies (V_Li_^−^) and anti-niobium (Nb_Li_^4+^) sites in LN crystals. The electronegativity of Li atoms, V_Li_^−^, Nb atoms, and Nb_Li_^4+^ increases in order, hence the electron density around N in the respective environment is improved. The nitrogen is the most promising element to substitute for the O site due to the similar atom radius and valence energy with that of the O atom [30]. Then the peaks of 392.3 eV and 394.1 eV possibly correspond to the bond of Li-N and the bond of V_Li_-N ions and the peaks of 395.9 eV and 398.1 eV were ascribed to Nb-N and Nb_Li_-N formed by N ions instead of the oxygen atoms, which is similar to N ions occupying the O sites in ZnO film doping N ions with binding energy of about 396.2 and 398.7 eV [31]. We can find that N ions were incorporated to the LN films with an N atom content of 0.87%.

Based on the results, it is reasonable to deduce that the N ions were successfully introduced into the LN films and located at the O sites, which form a shallow acceptor energy level. In addition, we can try to introduce other O-site dopants into LN to obtain p-type films that are indispensable for active devices of LN.

## 4. Conclusions

In summary, LN:N thin films have been successfully fabricated via a pulsed laser deposition with ideal conditions (70 Pa, 87 sccm, 18 mA) were also determined via a pulsed laser deposition. The film is well polycrystalline with the predominant orientations of along (012) and (104). The AFM of LN:N film indicates that the surface morphology is good and smooth, with a root mean-square roughness (RMS) of 3.12 nm. Futher, the Z range shows us the height being 16.76 nm. Most importantly, Hall-effect measurement demonstrates that LN:N thin films have p-type conduction. The holes concentration of the film is 7.31 × 10^15^ m^−3^ and the mobility is up to 266 cm^2^V^−1^s^−1^. Besides, XPS measurements indicates that N ions were successfully doped into LN films by PLD assisted RF and they likely substitute for O-sites to form a shallow energy level. This work provides a novel way to fabricate the p-type LN thin films, which will provide more choices and ideas for photoelectronic active devices based on the p-n junctions.

## Figures and Tables

**Table 1:** Fabrication conditions for deposition of LN:N thin films. The samples were fabricated with different air pressure, gas flow, and RF current.

Sample	Pressure (Pa)	Gas Flow (sccm)	RF Current (mA)
1#	43 N_2_	87	18
2#	53 N_2_	87	18
3#	70 N_2_	87	18
4#	70 N_2_	45	18
5#	70 N_2_	100	18
6#	70 N_2_	87	12
7#	70 N_2_	87	20
